# Cutaneous Inflammatory Malignant Fibrous Histiocytoma Presenting with a Leukemoid Reaction: A Case Report and Review of the Literature

**DOI:** 10.1155/2012/798629

**Published:** 2012-07-10

**Authors:** Jorge Hurtado-Cordovi, Boris Avezbakiyev, Marianne Frieri, Lester Freedman, Wondwoosen Gebre

**Affiliations:** ^1^Nassau University Medical Center, 2201 Hempstead Turnpike, East Meadow, NY 11554, USA; ^2^Division of Hematology/Oncology, Nassau University Medical Center and North Shore-Long Island Jewish Health System, East Meadow, NY 11554, USA

## Abstract

Malignant fibrous histiocytoma (MFH) is the most common sarcoma found in adults. We discuss a case of inflammatory MFH of dermal/epidermal origin presenting with a severe leukemoid reaction (LR). A 60 years old white male presented to hematology/oncology clinic complaining of mild shortness of breath on exertion. Past medical history was remarkable for removal of a left upper extremity necrotic mass 4.4 × 3 × 3 cm. Microscopy of the specimen showed clear surgical margin, and tumor cells restricted to the dermis without lymphovascular invasion. Immunohistochemestry was positive for CD 68 and CD 99. Chest x-ray was negative for metastatic disease. White blood cell count was 109.4 k/mm^3^ with 24 k/mm^3^ band neutrophils, and absolute neutrophil count of 69 k/mm^3^. CT scan of the thorax revealed numerous bilateral pulmonary nodules suspicious for metastasis. Based on these findings patient was diagnosed with metastatic cutaneous IMFH associated with a LR. Following review of medical literature, this appears to be the first reported case of inflammatory cutaneous MFH associated with LR. This histological variant is rare, and carries a poor prognosis. Thus, we would like to emphasize the need for investigating alternative therapies capable of improving the survival of these patients.

## 1. Introduction

Malignant fibrous histiocytoma (MFH) also known as undifferentiated pleomorphic sarcoma (UPS) is the most common soft tissue tumor of adults. It was first described by Kauffman and Stout [1]. Sarcomas are rare, and in the year 2005 they represented less than 1% of malignant tumors diagnosed in the United States. Thus, the exact incidence of MFH is unknown. This neoplasm usually develops in the six or seventh decade of life. MFH arises from a mesenchymal cell progenitor; it can develop as a novo lesion or secondary to radiation, surgery, osteonecrosis, Paget's disease, fibrous dysplasia, among others. It can affect virtually any part of the body, but it is most frequently found in the extremities, and up to 50% of them are located in the lower limbs. Patients frequently present complaining of a painful mass that has developed over a short period of time. Retroperitoneal tumors usually present with additional signs/symptoms of a space-occupying lesion. There are 5 morphological variants: storiform-pleomorphic which accounts for 70% of all cases, myxoid 20%, giant cell 10%, inflammatory 8%, and angiomatoid 2% [2–4].

Inflammatory malignant fibrous histiocytoma (IMFH) was initially identified as a separate category by Kyriakos and Kempson [4]. Its name is derived from its distinct histological characteristic, an extreme inflammatory infiltrate that may be composed of neutrophils, eosinophils, and/or lymphocytes without a recognized infection. This neoplasm is usually bulky, often recurs locally, and has a high metastatic potential. Elevated white blood cell counts (WBC) are common and are composed mostly of neutrophils, but eosinophilia has been described as well [5]. Patients typically present with fever and other constitutional symptoms mimicking an infectious process. Osaka et al. presented a case of MFH accompanied by prolonged spiking fevers which disappeared after tumor resection, and the fever was closely related to the clinical course of the illness. Expression of IL-8 mRNA was detected in preoperative peripheral blood mononuclear cells by reverse transcriptase-polymerase chain reaction (RT-PCR). Expressions of interleukin-6 (IL-6), IL-8, interferon (IFN)-gamma, and tumor necrosis factor (TNF)-alpha mRNAs were also detected in tumor tissue, while IL-1-alpha, IL-1-beta, IL-2, IL-4, and COX-2 mRNAs were not. Since infiltrating mononuclear cells as well as malignant cells were positive for IL-8, this may explain the tumor-associated fever. This observation is supported by other researchers' descriptions of fever resolution after surgical removal of the tumor and its reemergence once the malignancy has relapsed [4, 6, 7].

IMFH associated with leukemoid reaction is rare and its presentation as a destructive cutaneous lesion is also unusual [8]. Here we discuss a case of an aggressive inflammatory MFH of dermal/epidermal origin located on the left upper extremity presenting with a severe leukemoid reaction.

## 2. Case Report

**Figure 1:**
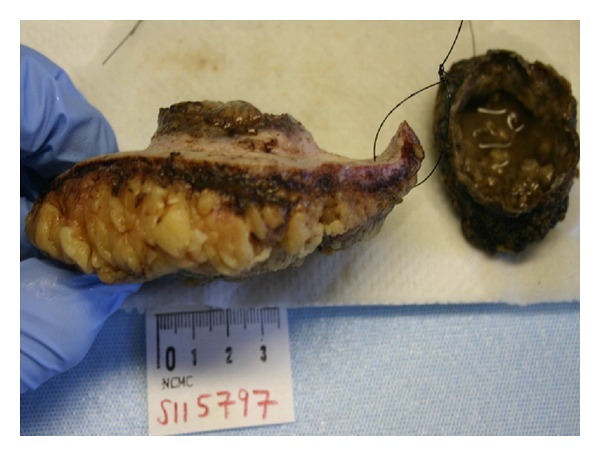
Gross specimen showing a section of skin with an ulcerative lesion (left) with necrotic tissue (right) that detached during resection.

**Figure 2:**
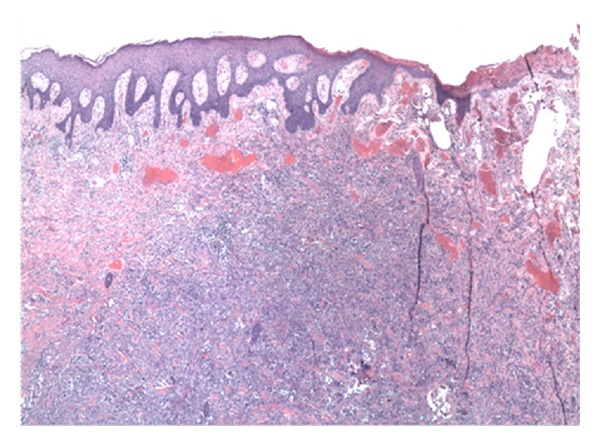
Section of skin showing malignant cells infiltrating the epidermis and dermis but without subcutaneous invasion (H&E 5x).

**Figure 3:**
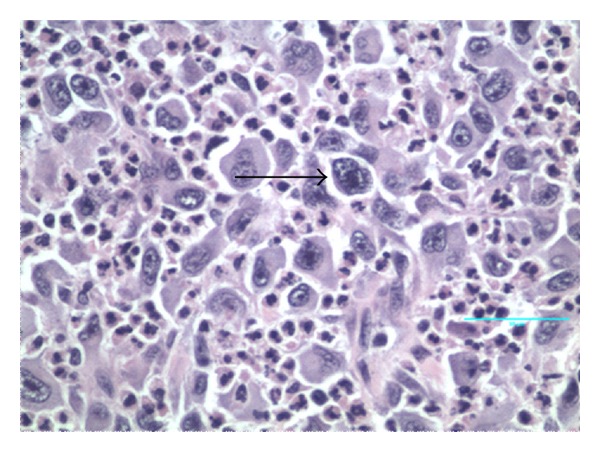
Slide showing giant malignant cells (arrow) in an inflammatory background (H&E) 40x.

**Figure 4:**
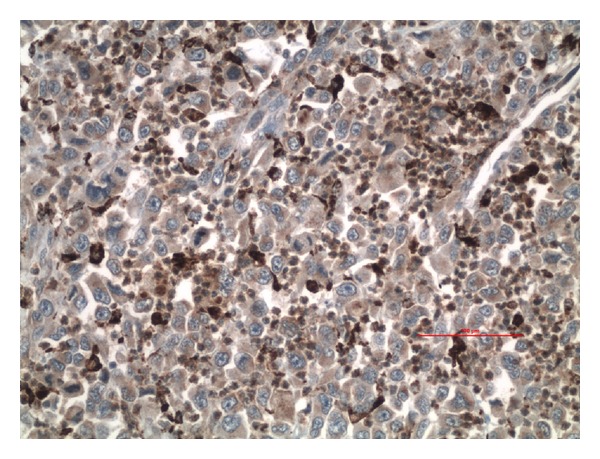
Malignant cells showing positive immunohistochemistry for CD 68 (20x).

**Figure 5:**
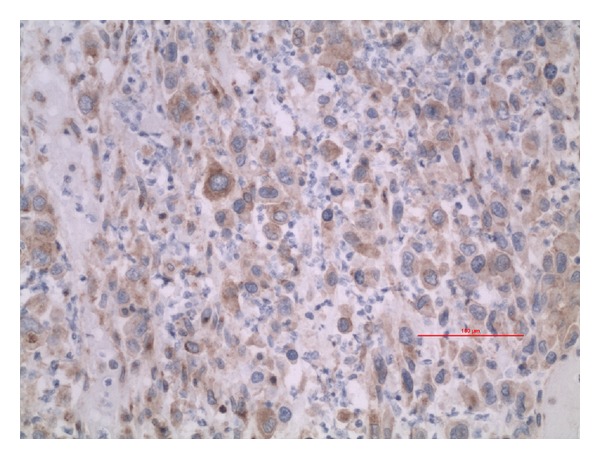
Malignant cells showing positive Immunohistochemistry for CD 99 (20x).

**Figure 6:**
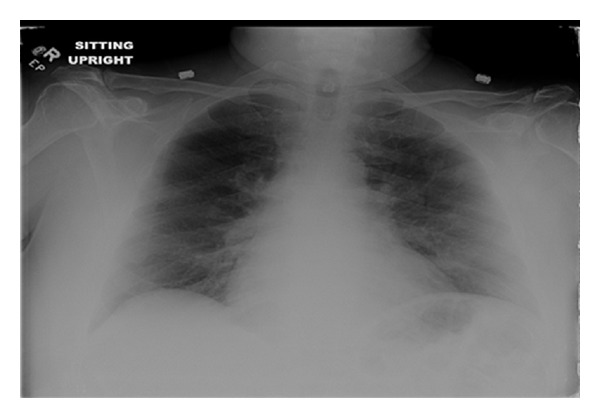
Presurgical chest radiography negative for metastasis.

**Figure 7:**
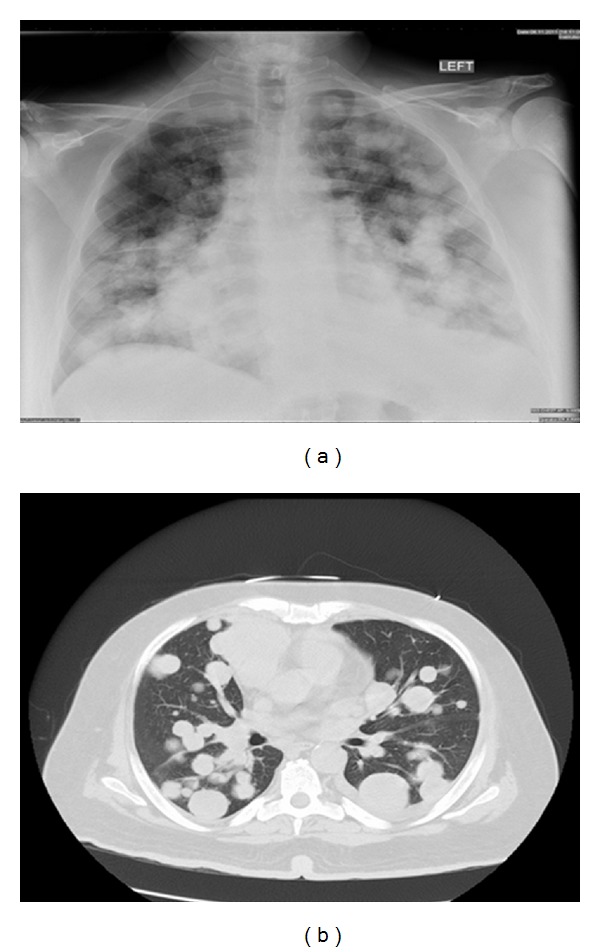
(a) Chest radiography showing multiple bilateral pulmonary nodules suggesting metastasis. (b) CT-scan of the chest revealing numerous pulmonary nodules suspicions for metastasis.

A 60-year-old white male presented to hematology/oncology clinic for a routine followup visit. The patient was complaining of mild shortness of breath on exertion. His past medical history consisted of hypertension and a left upper extremity necrotic mass, 4.4 × 3 × 3 cm, that was resected 5 weeks ago (Figure 1). Pathology revealed high-grade soft tissue undifferentiated sarcoma/pleomorphic malignant fibrous histiocytoma with clear resection margins tumor cells restricted to the dermis (Figure 2) without evidence of lymphovascular invasion. Giant malignant cells on an inflammatory background were identified on H&E stain (Figure 3.) Immunohistochemistry of the pathology specimen was positive for CD 68 and CD 99 (Figures 4 and 5) and negative for S-100, HMB-45, SMA, pan-keratin, factor 13-A, and desmin. At the time, a chest X-ray was negative for metastatic disease (Figure 6). A chest X-ray and CT scan of the thorax both revealed numerous bilateral pulmonary nodules (Figures 7(a) and 7(b)) suspicious for metastasis with left axillary and left superior diaphragmatic lymphadenopathy with splenomegaly and bilateral adrenal nodules. Routine blood work revealed a white blood cell count of 109.4 K/mm^3^ with absolute bands of 25 K/mm^3^ and an absolute neutrophil count of 64 K/mm^3^ (Table 1). C-reactive protein was 14.8 mg/dL. A peripheral blood smear showed presence of mature granulocytes with toxic granulations and vacuolizations and a significant left shift with excess of bands and occasional metamyelocytes and myelocytes. PCR for BCR-ABL fusion transcript was negative. His vital signs were as follows: pulse 123, BP 119/60, temp. 99.1°F, and RR 26–30. The patient was in mild respiratory distress. Head and neck examinations were unremarkable. Bilateral crackles were heard on lung auscultation. On cardiovascular exam, the patient was tachycardic, S1 and S2 were noted. The abdomen was soft, distended with mild diffuse tenderness, and present bowel sounds. Splenomegaly was noted on palpation. Blood cultures were drawn, which remain negative. Based on physical examination and data mentioned above, the patient was diagnosed with metastatic cutaneous IMFH associated with a leukemoid reaction. The patient was treated with supportive measures and broad spectrum antibiotics. Unfortunately, his breathing became increasingly labored, he failed several trials of BiPAP and was intubated. He remained tachypnic and became hypotensive. The patient did not receive vasopressors as per the family's wishes. The following day, the patient went into asystole and expired. Death occurred less than 45 days after initial diagnosis.

## 3. Discussion

IMFH is a rare soft tissue tumor. It can virtually affect any part of the body including solid organs such as the thymus, spermatic cord, and ovary. Most of them, however, arise from the retroperitoneal space [4, 9–11]. IMFH presenting as an ulcerative cutaneous lesion is extremely rare [8]. An elevated WBC is universally associated with this sarcoma; however, a leukemoid reaction with a leukocyte count above 50 K/mm^3^ is extraordinary [7, 12]. Thus, to the best of our knowledge this is the first time that an IMFH of cutaneous origin has been associated with severe leukemoid reaction.

IMFH develops from a mesenchymal cell progenitor [4]. However, there is a growing body of evidence that suggests that most retroperitoneal MFH, inflammatory and noninflammatory, is dedifferentiated liposarcomas. This theory is based on the findings that liposarcoma tissue has been identified within the tumor mass of neoplasms originally diagnosed as pure MFH, and the fact that these two entities share similar genomic profile, immunohistochemistry, and epidemiological distribution. Other experts suggest that the progenitor mesenchymal cells may retain their pluripotent capability and dedifferentiate into diverse cell lineages which could account for the presence of liposarcoma as well as other malignant tissues observed within MFH lesions. Thus, different sections of these tumors may represent different stages of dedifferentiation of the neoplasm [12–15]. After more than 30 years of being recognized as a separate entity, the natural history of this illness remains controversial.

At presentation, our patient's neoplasm was confined to the dermis with no evidence of lymphovascular invasion. Yet, advanced pulmonary metastasis was observed less than 40 days after resection despite a negative presurgical X-ray. This observation challenges the current view that superficial tumors have less metastatic potential and raises the question of which exact molecular mechanisms enable this tumor to rapidly metastasize [2, 12]. His clinical presentation was atypical. He originally came to the general medicine clinic because he noticed a rapidly growing mass in the left arm and wanted it removed for cosmetic reasons; hence, he never complained of any constitutional symptoms which are so characteristic of IMFH. Our case demonstrates that although IMFH usually presents with signs/symptoms mimicking infection, it can also manifest as an indolent cutaneous lesion. However, regardless of its clinical presentation, IMFH associated with leukemoid reaction carries a dismal prognosis [6, 12, 16, 17].

 The predominant malignant cells seen in IMFH are primitive macrophages and/or histiocytes that retain the capability of division and phagocytosis. They are also capable of manufacturing a variety of cytokines correlating with their cytologic origin [7]. This latter observation may explain the bone marrow hypercellularity, leukocytosis or leukemoid reaction, and other paraneoplastic symptoms, including fever, malaise, and weight loss seen in the majority of patients with IMFH. Different cytokines present in IMFH tumors appear to be responsible for the eosinophilic leukemoid reaction observed in one case and for the granulocytic leukemoid reaction observed in the other patient described by Melhem. They may also be responsible for expansion of the tumor-cell population, fibroblast proliferation, and enhanced secretion of extracellular collagen [7].

Research has shown that these cells can produce granulocyte colony stimulating factor (G-CSF), monocyte-granulocyte colony stimulating factor (GM-CSF), IL-6, IL-7, IL-8, stem colony factor (SCF), transforming growth factor beta (TGB), among others. Animal models have demonstrated that IL-6 can sustain neutrophil/macrophage colonies, and that through its action on other immune cells it can induce production of several colony stimulating factors by the bone marrow. IL-7 promotes leukocytosis by inducing proliferation of lymphocytes; it also increases WBC by stimulating secretion of IL-1, IL-6, and tumor necrosis (TNF) factor from circulating lymphocytes, monocytes, and macrophages. IL-8 is a powerful neutrophilic chemotactic and activating cytokine; thus, it may contribute to the intense inflammatory infiltrate seen in IMFH lesions. Under normal conditions, granulocyte production depends on the number of myeloid transcription factors as well as a broad range of substances that induce neutrophil release from the bone marrow including chemokines, cytokines, microbial products, and various other inflammatory mediators. Recent evidence on chemokine stromal derived factor-1 (SDF1, CXCL12) shows that they not only play a key role in neutrophil trafficking but are also involved in the pathways of cell survival, proliferation, and signaling involved in tumor progression, angiogenesis, metastases, and survival [7, 11, 18].

SCF combines with other grow factors and acts on the BM to increase the number of circulating erythrocytes, neutrophils, lymphocytes, monocytes, eosinophils, and basophils; it also contributes to the hypercellular BM observed in patients with IMFH associated with leukemoid reaction. Messenger RNAs for soluble as well as membrane bound SCF and its receptor tyrosine-protein kinase Kit (c-Kit) has been detected in MFH cell lines. It has been theorized that this growth factor may directly promote the development of these malignant cells through a paracrine mechanism via its c-Kit receptor. Other researchers argue that SCF may indirectly support proliferation of IMFH cells by promoting angiogenesis. In addition, it may be one of the biomolecules responsible for the aggressive nature of this malignancy [19, 20].

Yamamoto et al. investigated the expression of TGF-beta isoforms and their receptors in 43 human MFH specimens. This group found that all evaluated cases expressed different isoforms of this growth factor. Eighty-four percent of these cells expressed TGF beta receptor type 1 (R1), 56% expressed TGF beta R2, and 53% of them were positive for both. These researchers compared MIB-1 proliferation indices between MFH immunoreactive for both receptors with those that were positive for a single receptor. The MIB-1 index in the MFH group positive for both receptors was significantly higher than in the single positive group. This result suggests that the interactions of these receptors with their ligands have an important role in supporting the proliferation of these malignant cells through an autocrine and/or paracrine mechanism. Thus, further characterization of the TGB R1 and R2 coexpression may aid oncologists in predicting the behavior of this tumors [21].

In conclusion, increased production of these biomolecules by IMFH cells and their interaction with the inflammatory infiltrate seem to promote the development and growth of this neoplasm. This could also be responsible for the aggressive nature characterizing this illness. The elevated serum concentration of these cytokines could be responsible for most of the clinical signs/symptoms such as weight lost, anorexia, fever, and malaise, described by many patients when first seeking medical attention. Thus, more research is needed to understand the exact mechanism of how these proinflammatory cytokines and growth factors influence the development of these neoplastic cells. In addition, an understanding of how they manipulate the tumor's microenvironment favoring tumor growth should be further investigated in order to identify potential targets for therapy that could increase the overall survival of these patients. 

## Figures and Tables

**Table 1:** Display of the remarkable laboratory values during patient's hospitalization.

Date/time	WBC	Abs bands	ANC	Abs monocytes
November 8, 11:19 AM	109 K/mm^3^	25.2 K/mm^3^	64.5 K/mm^3^	12 K/mm^3^
November 8, 06:18 PM	106.6 K/mm^3^	21.3 K/mm^3^	77.8 K/mm^3^	3.2 K/mm^3^
November 9, 04:36 AM (after leukopheresis)	98.8 K/mm^3^	23.7 K/mm^3^	69.1 K/mm^3^	4 K/mm^3^
November 10, 01:46 AM	93.6 K/mm^3^	9.4 K/mm^3^	80.5 K/mm^3^	2.8 K/mm^3^
November 10, 04:46 AM (expiration day)	107.3 K/mm^3^	8.6 K/mm^3^	94.4 K/mm^3^	2.1 K/mm^3^

Abbreviations: WBC: white blood cell count, Abs bands: absolute band neutrophil count, ANC: absolute neutrophil count, Abs monocytes: absolute monocyte count, K/mm^3^: thousands per cubic millimeters.
